# Fracture Resistance of 3D-Printed Fixed Partial Dentures: Influence of Connector Size and Materials

**DOI:** 10.3390/ma18153468

**Published:** 2025-07-24

**Authors:** Giulia Verniani, Edoardo Ferrari Cagidiaco, SeyedReza Alavi Tabatabaei, Alessio Casucci

**Affiliations:** 1 Department of Prosthodontics, University of Siena, 53100 Siena, Italy; edoardo.ferrari.cagidiaco@gmail.com (E.F.C.); rezaalavi.taba@gmail.com (S.A.T.); 2 Division of Gerodontology and Removable Prosthodontics, University Clinics of Dental Medicine, University of Geneva, 1205 Geneva, Switzerland; alessiocasucci@gmail.com

**Keywords:** 3D printing, prosthodontics, fixed partial dentures, fracture resistance, connector size

## Abstract

Background: Limited data are available regarding the mechanical performance of 3D-printed fixed partial dentures (FPDs) fabricated from different materials and connector geometries. The purpose of this in vitro study was to evaluate the influence of connector size and material type on the fracture resistance of three-unit posterior FPDs fabricated with two commercially available 3D-printable dental resins. Methods: A standardized metal model with two cylindrical abutments was used to design three-unit FPDs. A total of sixty samples were produced, considering three connector sizes (3 × 3 mm, 4 × 4 mm, and 5 × 5 mm) and two different resins: Temp Print (GC Corp., Tokyo, Japan) and V-Print c&b temp (Voco GmbH, Cuxhaven, Germany) (n = 10). Specimens were fabricated with a DLP printer (Asiga MAX UV), post-processed per manufacturer recommendations, and tested for fracture resistance under occlusal loading using a universal testing machine. Data were analyzed using nonparametric tests (Mann–Whitney U and Kruskal–Wallis; α = 0.05). Results: Significant differences were found between material and connector size groups (*p* < 0.001). Temp Print (GC Corp., Tokyo, Japan) demonstrated higher mean fracture loads (792.34 ± 578.36 N) compared to V-Print c&b temp (Voco GmbH, Cuxhaven, Germany) (359.74 ± 131.64 N), with statistically significant differences at 4 × 4 and 5 × 5 mm connectors. Fracture strength proportionally increased with connector size. FPDs with 5 × 5 mm connectors showed the highest resistance, reaching values above 1500 N. Conclusions: Both connector geometry and material composition significantly affected the fracture resistance of 3D-printed FPDs. Larger connector dimensions and the use of Temp Print (GC Corp., Tokyo, Japan) resin enhanced mechanical performance.

## 1. Introduction

In recent years, advancements in additive manufacturing, particularly DLP and SLT 3D printing, have revolutionized the fabrication of dental prostheses, offering rapid production and cost-effectiveness [1,2]. Compared to subtractive manufacturing, 3D printing has a lower cost of production and allows less waste of materials [3]. As reported by Daher et al., 3D-printed composite resins are more cost-efficient at the production and equipment investment level compared to milling a pre-polymerized PMMA disk [4]. Additionally, the environmental sustainability of digital technologies is another point of interest [5]. In recent years, 3D printing has been increasingly adopted for the fabrication of interim restorations, and some studies have explored its application in long-term clinical scenarios as well [6,7,8]. These provisional crowns and fixed partial dentures (FPDs) play a critical role during the transitional phase between tooth preparation and placement of the definitive prosthesis [9,10]. Materials used for temporaries must ensure adequate esthetics, functional chewing ability, phonetic support, biocompatibility, protection of the abutment teeth, and sufficient mechanical performance [11]. In complex and multidisciplinary cases, the treatment plan may require the occlusion, function, and esthetics to be evaluated over an extended period with long-term interim restorations. These interim restorations can then serve as an accessible reference prototype for the final prostheses, which can be easily replicated using computer-aided design and computer-aided manufacturing (CAD-CAM) technology as final ceramic crowns or fixed partial dentures. The use of 3D printing for the fabrication of definitive fixed partial denture restorations remains limited [12], mainly due to the lower mechanical properties of the currently available 3D-printable materials. However, some in vitro studies have reported reduced fracture resistance in these materials compared to milled alternatives [13,14,15]. Recently, new materials with enhanced properties have been introduced to the market, offering greater potential for extended intraoral use [16]. These materials demonstrated improved occlusal stress dispersion and excellent durability under occlusal load [17,18]. For example, the recently released V Print c&b temp (Voco GmbH, Cuxhaven, Germany) shows, according to the manufacturer’s datasheet reports, a flexural strength of 132 MPa and a modulus of elasticity of 4417 Mpa. These values are notably higher than those reported for conventional polymethyl methacrylate (PMMA)-based materials [19]. The measured flexural strength values for the tested resins ranged from 113.37 ± 31.93 MPa to 156.56 ± 25.58 MPa, which are well above the minimum requirement of 80 MPa established by the ISO 4049:2019 standard for polymer-based restorative materials [20,21]. In a recent in vitro study simulating 60,000 chewing cycles, the 3D-printed resin exhibited significantly lower wear volume loss (0.10 ± 0.01 mm^3^) compared to both milled resin (0.21 ± 0.02 mm^3^) and conventional resin (0.44 ± 0.01 mm^3^) (*p* < 0.001). Additionally, the mean surface roughness (Ra) after simulation was lowest for the 3D-printed group (0.59 ± 0.06 µm), indicating a smoother surface finish post-wear when compared to milled (1.27 ± 0.49 µm) and conventional materials (1.64 ± 0.44 µm) (*p* = 0.01), supporting the promising performance of these new materials in terms of mechanical durability and surface integrity [22]. FPDs, whether provisional or definitive ones, are subjected to various stresses within the oral cavity, including chewing forces and parafunctional habits. Flexural strength is a critical mechanical property for dental prostheses, as it reflects the material’s ability to resist deformation and fracture under bending forces [23,24,25]. The fracture resistance of the FPDs is influenced by both the material’s composition and the structural design of the prosthesis. The importance of connector size is particularly pronounced in dental bridges, where the connector must support the pontic and resist occlusal forces during function. Insufficient connector dimensions can lead to stress concentration and eventual fracture, compromising the prosthesis’s longevity [26,27,28,29]. In fact, restorations with a lower fracture resistance may exhibit greater deformation under load, potentially leading to micromovements at the adhesive interface. These micromovements can contribute to the degradation of the bond over time, especially under cyclic loading conditions typical in the oral environment [29]. Therefore, understanding the relationship between connector size and fracture resistance is essential for designing durable and reliable dental restorations.

The null hypotheses tested in this in vitro study were the following:

1. Fracture resistance of FPDs is not influenced by the different 3D-printed material used.
2. Fracture resistance of 3D-printed FPDs is not influenced by the connector size of the restoration.
3. There is no interaction between material type and connector size that affects the fracture resistance of 3D-printed FPDs.

## 2. Materials and Methods

**Figure 1:**
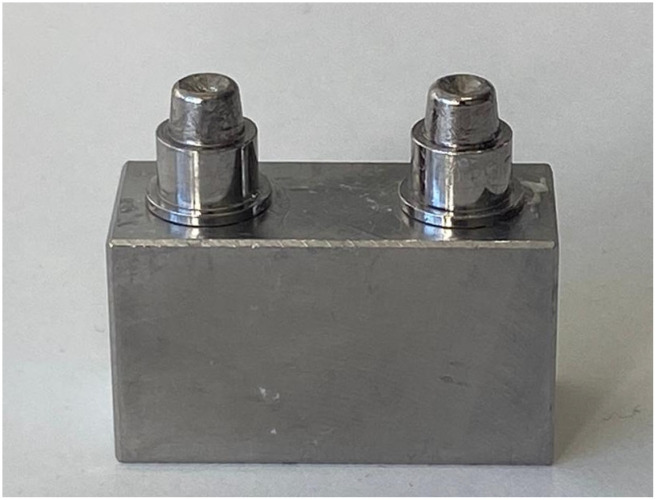
Steel reference model imitating a second premolar and a second molar with a distance of 16.5 mm between the 2 cylindrical abutments (diameter 7 mm with a 1 mm circular shoulder and a taper of 6 degrees).

A steel abutment model fabricated in AISI 440C martensitic stainless steel, imitating two cylindrical abutments (7 mm diameter, with a shoulder preparation) 16.5 mm from each other (Figure 1), was used in the present study to standardize the testing procedure as reported in previous studies [30].

**Figure 2:**
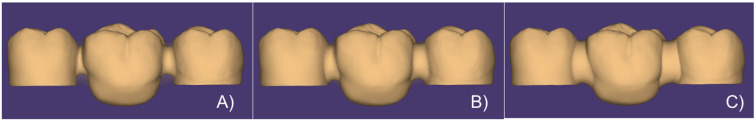
Stl files with different connector sizes: (**A**) 3 × 3; (**B**) 4 × 4; (**C**) 5 × 5.

The steel reference model was scanned using an intraoral scanner, Trios 4 (3Shape, Copenhagen, Denmark), and the produced standard tessellation file (.stl) was sent to the lab. An experienced lab technician designed a full-anatomic three-unit FDP (DentalCAD version 2.2, exocad GmbH, Darmstadt, Germany) and then finalized the project into 3 different .stl files, changing only the rectangular cuboid cross-section connector size into 3 × 3, 4 × 4, and 5 × 5, as shown in Figure 2.

**Figure 3:**
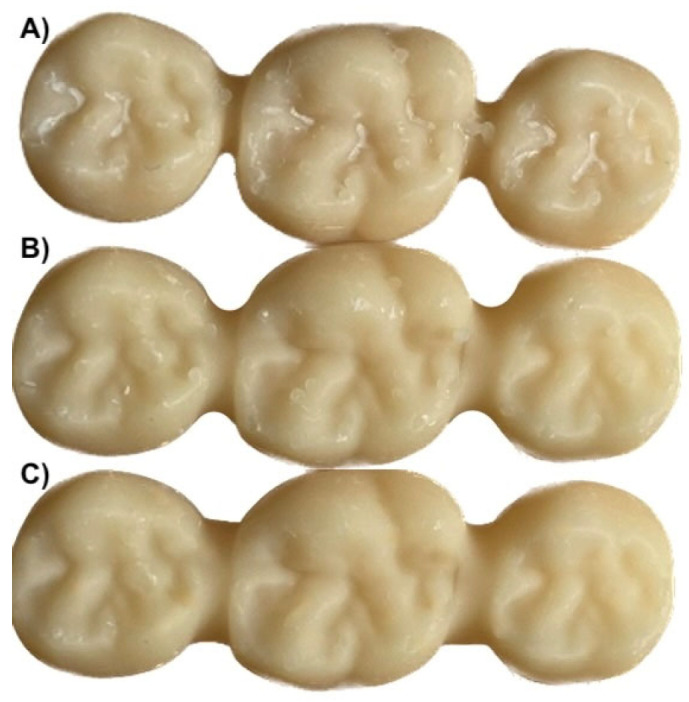
Samples that were 3D-printed with different connector sizes: (**A**) 3 × 3; (**B**) 4 × 4; (**C**) 5 × 5.

Two different 3D-printable materials were tested in the present study: (1) V-Print c&b temp (Voco GmbH, Cuxhaven, Germany) and (2) Temp Print (GC Corp., Tokyo, Japan), as reported in Table 1. A total of 60 specimens were tested: 10 specimens for each material and each connector size. A sample size of 10 per group was estimated to provide over 80% power to detect differences greater than 250 N at a significance level of α = 0.05. A digital light processing (DLP) printer, Asiga MAX UV (wavelength = 385, pixel resolution = 62; NSW, Hawthorn, Australia), was used to print the specimens at a 0-degree orientation to the build plate featuring a UV light-emitting diode with a wavelength of 405 nm and a printing layer thickness set at 100 μm. A slow-speed rotary tool was utilized to remove the support structures from the specimens. Then, Liquidtech BT was used to clean the samples for 20 min using the BB Wash machine (Meccatronicore S.R.L., Pergine Valsugana, TN, Italy). All the samples were polymerized with a BB cure machine (Model MTC-BB-CURE-COMPACT, Meccatronicore S.R.L., Pergine Valsugana, TN, Italy) for 40 min. Before testing, all the specimens were visually inspected following a uniform design, and the fit on the metal model was verified with an explorer. The final 3D-printed specimens with different connector sizes before testing are shown in Figure 3.

**Figure 4:**
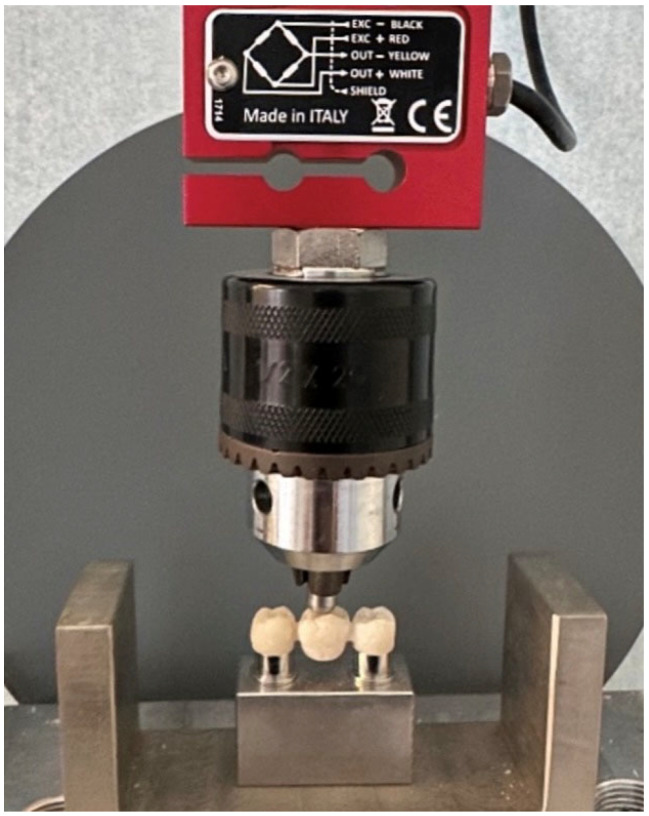
Universal machine for three-point bending test.

**Figure 5:**
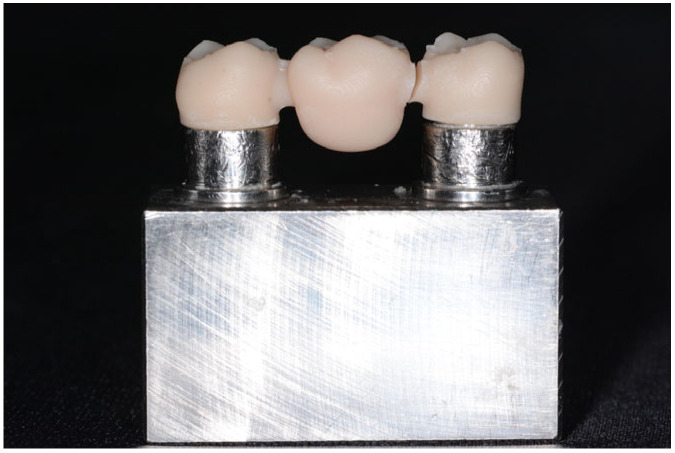
Specimen after fracture.

All specimens were tested in a universal testing machine (5567 Universal Testing Machine; Instron Ltd., Norwood, MA, USA) with a 500 N cell load. The fracture test was carried out by means of the load compression mode occlusally applied to the center of each specimen on the pontic surface using a 3 mm diameter metal sphere at a crosshead speed of 5 mm/min until failure occurred, as shown in Figure 4 and Figure 5. The maximum of the fracture force was recorded in Newtons. No luting agent was used to bond the FDP to the abutments of the metal model.

Statistical analysis was performed using the Statistical Package for the Social Sciences (SPSS) software, version 26 (IBM Inc., Armonk, NY, USA). After the Kolmogorov–Smirnov test, the Mann–Whitney U test and the Kruskal–Wallis tests were used to compare the fracture load between the different groups. The significance level was considered at *p* < 0.05.

## 3. Results

The normality of the data was assessed using the Kolmogorov–Smirnov test, which indicated a non-normal distribution for fracture load values (*p* < 0.001). Consequently, nonparametric tests were applied.

The results of the fracture resistance test revealed statistically significant differences between materials and connector sizes. Temp Print (GC Corp., Tokyo, Japan) exhibited a significantly higher mean fracture load (792.34 ± 578.36 N) compared to V-Print c&b temp (Voco GmbH, Cuxhaven, Germany) (359.74 ± 131.64 N) as reported in Table 2. However, the difference was statistically significant only for connector sizes 4 × 4 and 5 × 5 (*p* < 0.001), while no statistically significant difference was observed for connector size 3 × 3 (*p* = 0.414) as reported in Table 3. The fracture resistance was positively influenced by the increase in connector size across all groups, as reported in Table 3. The mean fracture loads were 254.72 ± 40.65 N for 3 × 3 connectors, 464.07 ± 130.00 N for 4 × 4 connectors, and 1037.99 ± 589.93 N for 5 × 5 connectors. The Kruskal–Wallis test revealed a statistically significant difference between groups (*p* < 0.001).

**Figure 6:**
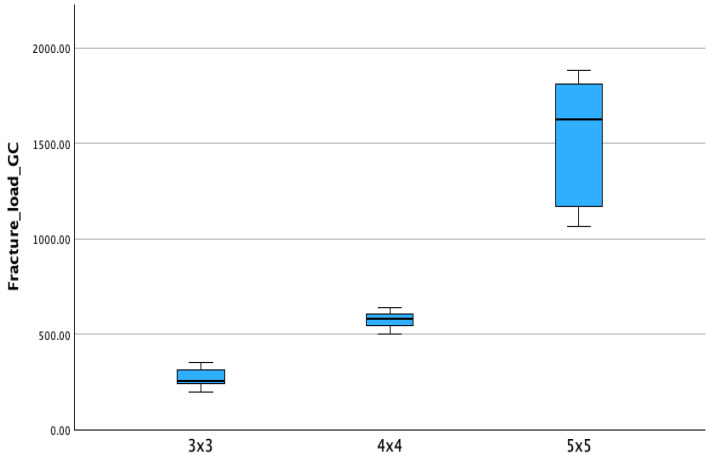
Boxplots of fracture resistance (N) for Temp Print (GC Corp., Tokyo, Japan) with different connector sizes (3 × 3 mm, 4 × 4 mm, 5 × 5 mm). Boxes show data distribution with median and whiskers.

**Figure 7:**
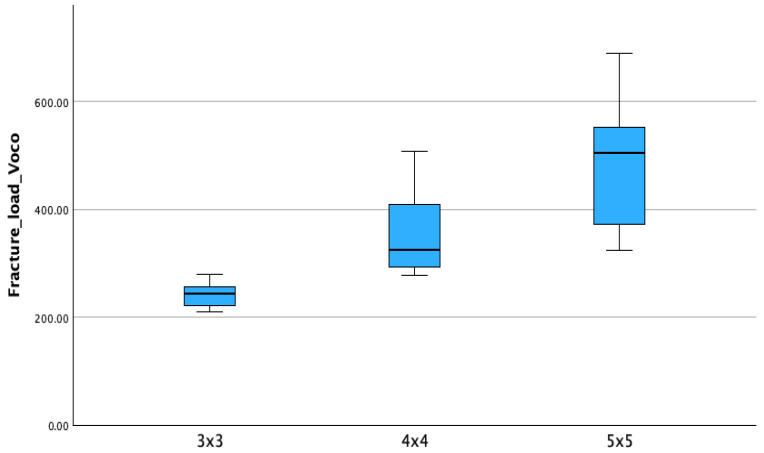
Boxplots of fracture resistance (N) for V-Print c&b temp (Voco GmbH, Cuxhaven, Germany) with different connector sizes (3 × 3 mm, 4 × 4 mm, 5 × 5 mm). Boxes show data distribution with median and whiskers.

Within the Temp Print group, the increase in connector size resulted in higher fracture strength as reported in Table 4. Mean values for Temp Print (GC Corp., Tokyo, Japan) were 266.09 ± 50.79 N (3 × 3), 576.82 ± 41.72 N (4 × 4), and 1534.12 ± 320.68 N (5 × 5). Similarly, V-Print c&b temp (Voco GmbH, Cuxhaven, Germany) also showed an increase in strength with larger connector sizes, although absolute values remained lower. Boxplots of fracture load values for each material are shown in Figure 6 and Figure 7.

## 4. Discussion

The results of the present in vitro study demonstrated that both connector size and resin type significantly affected the fracture resistance of 3D-printed posterior fixed partial dentures (FPDs); thus, all the null hypotheses were rejected. An increase in connector dimensions corresponded with a substantial enhancement in fracture resistance, and among the materials tested, Temp Print (GC Corp., Tokyo, Japan) resin exhibited superior mechanical performance compared to V-Print c&b temp (Voco GmbH, Cuxhaven, Germany) resin. The influence of connector size on fracture resistance is well-documented in the prosthodontic literature and can be attributed to the fundamental principles of biomechanics [26,27,28]. The complexity of the prosthesis design, such as connector thickness and the distribution of material stress points, could lead to differing results in fracture resistance tests [24]. According to basic biomechanical principles, the section modulus of a structure—which determines its resistance to bending—is directly proportional to the height and width of the connector [31]. Larger connectors possess higher stiffness, which leads to decreased flexural deflection under load and a more uniform distribution of stress across the connector region [32]. As a result, prostheses with larger connector dimensions exhibit greater fracture resistance and longevity, particularly under posterior occlusal forces. From a clinical perspective, when using 3D-printed materials to realize a long-term interim FPD, the clinician and the lab technician should take into consideration using a larger connector, such as 5 × 5, even if there is a compromise in the esthetics of the restoration.

Material selection further influenced the fracture behavior of the 3D-printed FPDs. Specimens fabricated using Temp Print (GC Corp., Tokyo, Japan) resin showed significantly greater fracture load values across all connector sizes compared to those made with V-Print c&b temp (Voco GmbH, Cuxhaven, Germany) resin. The superior performance of Temp Print (GC Corp., Tokyo, Japan) may be attributed to differences in resin formulation and filler content that contribute to enhanced mechanical properties [33,34]. These results underscore the importance of material-specific characteristics in the performance of additively manufactured interim restorations and caution against assuming interchangeability among commercially available resins. The fracture resistance values obtained in the present study (359.74 ± 131.64 N) were higher than those reported by Greuling et al. for 3D-printed four-unit FPDs (294 ± 88.3 N), likely due to differences in restoration design—especially span length—as well as differences in material selection [30]. Some values in our study were, however, lower than those obtained by Reymus et al. [35]. In fact, while a fracture resistance of 1050.4 ± 133.3 N was reported for NextDent C&B, in the present study, Temp Print registered 576.82 ± 41.72 N, while V-Print registered 351.32 ± 75.41 N with comparable connector sizes. However, the findings of this study differ from those reported by Ibrahim [36], who documented considerably higher fracture resistance values for 3D-printed provisional restorations, with some reaching up to 1226.48 N, outperforming milled restorations under comparable testing parameters. Despite the controlled testing conditions, variability in fracture strength may reflect intrinsic differences in the internal structure of the printed resins or limitations inherent to the additive manufacturing process, such as interlayer adhesion defects or different post-curing protocols [37]. Clinical occlusal forces typically range from 400 to 800 N, with peak forces in the posterior regions occasionally reaching up to 1500 N [38,39,40]. These values depend on various factors such as the measurement method, patient gender, type of restoration, dietary habits, and other individual parameters [38,39]. Thus, the fracture load tested in the present study may not withstand the clinical applications for definitive restorations. In fact, flexural behavior is closely tied to material properties. Co-Cr alloys and zirconia offer high stiffness (Young’s modulus ≥ 200 GPa) and flexural strength > 800 MPa [40,41], making them ideal for definitive restorations. In contrast, 3D-printed resins like Temp Print and V-Print C&B Temp show lower elastic modulus (2000–4500 MPa) and strength (100–130 MPa), which may limit their use in permanent restorations but render them appropriate for short- to medium-term interim FPDs [42]. The cost ratio of 3D-printed resin-based materials may offer advantages in certain clinical contexts; however, this benefit must be weighed against the potential for increased deflection, wear, and long-term dimensional instability as reported in the high mechanical failures reported in the 3-year RCT conducted by Hobbi et al. [43]. Nevertheless, these materials are widely applicable and suitable for use as interim restorations. In fact, previous studies have reported flexural strength values for conventional PMMA-based acrylic interim FPDs ranging between approximately 80 and 250 MPa, depending on formulation and testing conditions. In terms of fracture load, values for conventionally fabricated acrylic FPDs are often below 500–600 N, especially in long-span restorations [44,45].

A limitation of this study is the use of a rigid steel model, which may have influenced the fracture load results by reducing the compliance typically present in clinical conditions [46]. Moreover, the FDPs were not cemented onto the abutments, possibly resulting in altered load distribution and diminished energy absorption. Future studies should investigate the effects of cementation and model flexibility on fracture resistance. Additionally, deflection data and displacement recording should be registered. Another limitation of this study was the use of uniaxial loading at the central connector, which may not accurately reflect clinical conditions, as human mastication involves multidirectional forces and varies among individuals. Additionally, surface roughness and hardness were not measured, which may affect stress distribution and fracture behavior.

From a clinical perspective, the findings of this study highlight the necessity of careful consideration of both connector design and resin selection when fabricating 3D-printed interim FPDs. While increasing connector dimensions may improve mechanical performance, such design modifications must be reconciled with clinical constraints. Moreover, the choice of resin should be guided not solely by printability or workflow efficiency, but by validated mechanical properties appropriate to the clinical indication.

## 5. Conclusions

Within the limitations of this in vitro study, the following conclusions can be drawn:

1. Both material composition and connector size significantly influenced the fracture resistance of 3D-printed posterior fixed partial dentures.
2. Temp Print (GC Corp., Tokyo, Japan) resin exhibited superior mechanical performance compared to V-Print c&b temp (Voco GmbH, Cuxhaven, Germany) across all connector dimensions, indicating that resin formulation plays a key role in fracture resistance.
3. Increasing the connector size from 3 × 3 mm to 5 × 5 mm led to a substantial improvement in fracture load, with the largest connectors yielding the highest strength values.
4. Careful selection of both material and connector design is essential for the clinical success of 3D-printed interim fixed partial dentures, especially in high-load posterior regions.

## Figures and Tables

**Table 1:** Materials tested in the present study.

Product Name	Manufacturer	Components	Batch	Properties Provided by the Manufacturer
Temp PRINT	GC Corp., Tokyo, Japan	Urethane dimethacrylate (UDMA) dimethacrylate component quartz (SiO_2_) photoinitiator synergist UV-light absorber.	10004797	Viscosity 0.5–2.0 Pa s; density 1.1–1.3 g/mm^3^; flexural strength > 90 MPa; sorption < 40 µg/mm^3^; solubility < 7.5 µg/mm^3^; layer thickness 50 µm; light wavelength 385–405 nm.
V-Print c&b temp	Voco GmbH, Cuxhaven, Germany	UDMA Bis-EMA TEGDMA 50–100% 25–50% 5–10% 4,4-Isopropylidenediphenol, ethoxylated 2-methylprop-2enoic acid, benzeneacetic acid, alpha.	6897	Viscosity 2.80 Pa s; flexural strength 132 MPa (DIN EN ISO 10477); modulus of elasticity 4417 MPa (DIN EN ISO 178); water absorption 17.63 µg/mm^3^ (DIN EN ISO 10477); water solubility 0.68 µg/mm^3^ (DIN EN ISO 10477); filler content 26% by weight.

**Table 2:** Mean fracture resistance (mean), standard deviations (SDs), and significant differences (Sign.) for each material.

Material	Mean (N) ± SD	Sign.
Temp Print	792.34 ± 578.36	A
V-Print	359.74 ± 131.64	B

Different letters indicate statistically significant differences (*p* < 0.05).

**Table 3:** Mean fracture resistance (mean), standard deviations (SDs), and significant differences (Sign.) for different connector sizes.

Connector	Mean (N) ± SD	Sign.
3 × 3	254.72 ± 40.65	C
4 × 4	464.07 ± 130.00	B
5 × 5	1037.99 ± 589.93	A

Different letters indicate statistically significant differences (*p* < 0.05).

**Table 4:** Mean fracture resistance (mean), standard deviations (SDs), and *p*-values for each material and connector size.

Connector	Temp Print (N) ± SD	V-Print (N) ± SD	*p*-Value
3 × 3	266.09 ± 50.79 A,a	242.09 ± 21.81 A,a	0.414
4 × 4	576.82 ± 41.72 B,a	351.32 ± 75.41 B,b	<0.001
5 × 5	1534.12 ± 320.68 C,a	486.74 ± 133.43 C,b	<0.001

Statistically significant differences are highlighted with different letters (*p* < 0.05); capital letters are used to highlight statistically significant differences between columns, and lowercase letters are used for statistically significant differences between rows.

## Data Availability

The data presented in this study are available on request from the corresponding author. The data are not publicly available due to university policy.
